# Patient perceptions of the benefits and barriers of virtual postnatal care: a qualitative study

**DOI:** 10.1186/s12884-021-03999-9

**Published:** 2021-08-07

**Authors:** Megan Saad, Sophy Chan, Lisa Nguyen, Siddhartha Srivastava, Ramana Appireddy

**Affiliations:** 1 School of Medicine, Queen’s University, Kingston, ON Canada; 2 Department of Medicine, Queen’s University, Kingston, ON Canada

**Keywords:** Telemedicine, Postpartum Period, Postnatal Care, Qualitative Research, Social Determinants of Health

## Abstract

**Objective:**

The objective of this study is to understand the perceptions of new mothers using virtual care via video conferencing to gain insight into the benefits and barriers of virtual care for obstetric patients.

**Methods:**

Semi-structured interviews were conducted with 15 patients attending the Kingston Health Sciences Centre. The interviews were 20–25 min in length and recorded through an audio recorder. Thematic analysis was conducted in order to derive the major themes explored in this study.

**Results:**

New mothers must often adopt new routines to balance their needs and their child’s needs. These routines could impact compliance and motivation to attend follow-up care. In our study, participants expressed high satisfaction with virtual care, emphasizing benefits related to comfort, convenience, communication, socioeconomic factors, and the ease of technology use. Participants also perceived that they could receive emotional support and build trust with their health care providers despite the remote nature of their care. Due to its ease of use and increased accessibility, we argue that virtual care shows promise to facilitate long-term compliance to care in obstetric patients.

**Conclusions:**

Virtual care is a useful modality that could improve compliance to obstetric care. Further research and clinical endeavours should examine how social factors and determinants intersect to determine how they underpin patient perceptions of virtual and in-person care.

**Supplementary Information:**

The online version contains supplementary material available at 10.1186/s12884-021-03999-9.

## Introduction

A significant proportion of women experience medical complications such as hypertension and diabetes in the postpartum period [1, 2]. Many of these conditions require and benefit from ongoing medical care, as long-term prognosis could lead to an elevated risk of cardiovascular disease and mortality [1–3]. Hypertensive disorders and gestational diabetes have been associated with a 50–300% increase in risk for cardiovascular disease [4, 5]. A multitude of factors related to pregnancy and associated medical complications can significantly affect the quality of life of new mothers [6–9]. Thus, clinical intervention in the postpartum period is significant in reducing maternal morbidity and increasing quality of life. However, the burden and demands of dealing with pregnancy and the post-partum period along with the pre-existing barriers (socio-demographic and geographical) in accessing health care can negatively impact patient compliance in attending postnatal follow-up clinics [10, 11]. Telemedicine can improve accessibility to postnatal healthcare services for this unique patient population and thus enhance patient care [12, 13]. Virtual care allows patients to consult with their healthcare provider through digital technologies. Virtual care can be conducted through various modalities, such as email, text messaging, or audio-videoconferencing and through personal digital devices, such as a smartphone or tablet, without any additional infrastructure required [14–16].

The World Health Organization defines the social determinants of health as the conditions in which people are born, grow, work, live, and age, and the wider set of forces and systems shaping the conditions of daily life [17]. The Social Determinants of Health (SDoH) framework posits that biological outcomes and access to health care are mediated by social inequalities [18]. In particular, gender uniquely intersects with other social and environmental determinants. As such, new mothers are often burdened by a unique set of challenges and can be disproportionately limited in their access to health care. Immigration status, language proficiency, Aboriginal status, food insecurity, poverty and rurality are known determinants that make it difficult for mothers to obtain the care they need [19]. However, the SDoH may uniquely impact health and healthcare access for new mothers given that considerations around accessibility may be different. Therefore, the objective of this study was to understand the perspectives of new mothers using virtual visits for postnatal follow-up care to gain insight into benefits and barriers of virtual care for obstetric patients. These insights can elucidate whether virtual care can address challenges related to health care access.

## Methods

### Study Design

This paper draws on a case study research design. A case study approach allows for an in-depth exploration of complex issues or a phenomenon in a given context and explores the multiple aspects of a phenomenon through the perspectives, descriptions, and lived experiences of the persons impacted by the issue or phenomenon at hand [20, 21]. The SAGE Encyclopedia of Qualitative Research defines perception as “a mode of apprehending reality and experience through the senses” [22]. Researchers capture perspectives through narratives that detail life experiences, behaviours, and reactions. Through an in-depth analysis of perceptions, researchers can understand how different factors determine an individual’s experiences of a phenomena. Thus, we are using a case study research design to provide readers with a generalizable, but also complex, portrayal of how patients experience and perceive virtual care in an obstetric context. By asking participants about their experiences with postnatal virtual care, we sought to elucidate the perceptions of participants as to the benefits and barriers of this modality of care compared to traditional in person visits.

To capture these nuanced perspectives, we conducted semi-structured interviews with patients being seen at the Kingston Health Sciences Centre (KHSC) for maternal postnatal follow-up virtual visits with an obstetric medicine physician. The purpose of semi-structured interviews is to guide interviewees to speak openly about their experiences and perspectives accessing postnatal virtual follow-up healthcare appointments without any pre-set answers [23]. The open-ended nature of the questions also let participants to draw on different aspects of their experiences to inform their perspectives.

### Research Setting

The Kingston Health Sciences Centre (KHSC) is the largest acute-care academic hospital in south-eastern Ontario, the most populated province in Canada. The KHSC consists of the Kingston General Hospital and Hotel Dieu Hospital and sees over 500,000 patients from the region. The obstetrics program at KHSC is carried out at the Kingston General Hospital and is the largest obstetrics program in southeastern Ontario. The obstetric care team supports childbearing persons with out-patient care and provides educational services to support child-bearing persons, particularly individuals presenting with comorbidities, throughout and following pregnancy. The research team is comprised of a medical student, two obstetric medicine physicians, and two virtual care researchers who seek to better understand and improve the virtual care experience for all patients. To prevent bias, only the medical student and researcher members conducted interviews with the participants. The obstetric physician members recruited the participants and provided crucial context for the project.

### Sampling Strategy

The research team employed a purposive sampling strategy. New mothers who completed at least one virtual visit appointment at the Kingston General Hospital were invited to participate in the study. This was determined by the obstetric physician member on our study team. All prospective participants were required to speak conversational English and be over the age of 18. The virtual visits, conducted via video conferencing, were hosted through the Ontario Telemedicine Network (OTN) [24]. A medical secretary and two members of the research team invited all the participants to the study by telephone. A Letter of Information was sent through email to all interested participants. In the Letter of Information, the participants were let known that the research team sought to improve patient experience using virtual care and to understand their perspectives of receiving health care through virtual care.

### Data Collection

Thirty-one patients who completed a virtual visit appointment between February and August 2020 were invited to participate in this study [23]. A medical secretary invited all potential participants by telephone. Two study team members (S.C. and M.S.), a research associate and a medical student, conducted the telephone interviews. The interview tool was developed in English drawing on similar survey tools, relevant literature, and patient-oriented interview guides employed by our team in previous projects. Due to the COVID-19 pandemic, the interviews were conducted over the telephone and recorded using an audio recorder. With sensitivity to the participant’s availability, all interviews lasted 20–25 min. The interviews were aimed to be kept short and concise considering the participant demographic, as all of the interviewees were new mothers who had just recently given birth and were concurrently caring for their children at the time of the interviews. Further, many of our participants only agreed to participate in the study if the interview timeframe was relatively short due to the need to look after their dependents. Field notes were taken during the interviews.

### Data Analysis

The interviews were transcribed verbatim by a hired transcriptionist, and the transcripts coded by S.C. and M.S. The data was coded using NVivo 12.0. Braun and Clarke’s thematic analysis approach was used throughout the coding process for identifying, analyzing and reporting patterns within the data [25]. Consensus of the coding themes was reached through an iterative process, where S.C. and M.S. came together at every 5^th^ stage of the coding process to review and refine the parent themes and subthemes.

We determined that saturation was achieved when 1) the same themes were repeated by participants, 2) no new themes emerged from the data, and 3) when no other eligible individuals could be recruited within the study period. All individuals who fit the eligibility criteria and were seen by obstetric physician team members on this project between March and August 2020 were contacted to participate in this study. This time period was chosen to ensure that only new mothers up to 4-months post-partum could participate in this study.

## Results

Out of thirty-one potential participants, fifteen participants were interviewed from July–August 2020. Six patients declined to participate in the study. Nine participants signed up to participate but did not end up completing the interview following multiple attempts at follow-up. It was decided to recruit and interview a limited number of participants to maintain a strong level of rigor in the data collection and analysis. The ages of participants ranged from 22 to 41 years. All participants identified as female, and majority of participants were married, Canadian-born and of white/Caucasian ethnicity. A majority of participants were also employed in some capacity, living in Kingston, ON, and had a bachelor’s degree or above (Table 1). Details on travel distance, travel time, number of virtual care visits, and the medical profiles of the participants are listed in Table 2.

**Table 1:** Sociodemographic data of all participants (*n* = 15)

Variable	Values	Number (%)
**Age categories**	20–24	1 (7%)
	25–29	2 (13%)
	30–34	6 (40%)
	35–39	4 (27%)
	40–44	2 (13%)
**Gender**	Female	15 (100%)
	Male	0 (0%)
**Ethnicity**	White/Caucasian	8 (53%)
	South Asian	1 (7%)
	African	1 (7%)
	Portuguese	1 (7%)
	Arab	1 (7%)
	Unknown	3 (20%)
**Employment**	Healthcare Sector	6 (40%)
	Education Sector	2 (13%)
	Administration	2 (13%)
	Creative Professional	1 (7%)
	Student	1 (7%)
	Unemployed	1 (7%)
	Unknown	2 (13%)
**Education**	High School	1 (7%)
	College Diploma	2 (13%)
	Bachelor’s Degree	6 (40%)
	Master’s Degree	2 (13%)
	PhD	1 (7%)
	Unknown	3 (20%)
**Place of Residence**	Urban (in Kingston, ON)	5 (33%)
	Urban (Outside Kingston, ON)	4 (27%)
	Rural (Outside Kingston, ON)	6 (40%)
**Household Income**	$0 to $30,000	3 (20%)
	$30,000 to $59,999	2 (13%)
	$60,000 to $89,999	3 (20%)
	$90,000 to $119,999	3 (20%)
	$120,000 to $149,999	0 (0%)
	$150,000 or more	1 (7%)
	Unknown	3 (20%)
**Marital Status**	Married	13 (87%)
	Unmarried (in a relationship)	1 (7%)
	Unmarried (single)	1 (7%)
**Immigrant Status**	Canadian-Born	9 (60%)
	Non-Canadian Born	3 (20%)
	Unknown	3 (20%)

**Table 2:** Travel time, number of virtual visits, and medical profiles of participants

Patient	Age	Two-way travel Distance (km)	Two-way travel time (min)	Number of virtual visits	Gravida-para/TPAL status	Pregnancy status	Medical conditions
Patient 1	36	160	132	1	G4T1A2L1	Postpartum	Pregnancy related pulmonary embolism
Patient 2	35	44.8	50	3	G2T2	Postpartum	Hypertension
Patient 3	34	118	162	1	G2P1A1	Postpartum	Gestational hypertension
Patient 4	34	36.2	48	1	G2 P2	Postpartum	Hypertension; gestational diabetes; mental health disorder;
Patient 5	34	7.4	14	3	G1 P1	Postpartum	Preeclampsia; hypertension; iron deficiency anemia
Patient 6	34	145.4	108	2	G2P3 (G1 was twins)	Postpartum	Postpartum hypertension; tachycardia; preeclampsia;
Patient 7	32	6.6	12	2	G3P2A1	Postpartum	Chronic hypertension; Bell’s palsy; proteinuria;
Patient 8	31	6	10	1	G2P2	Postpartum	Hypertension, celiac disease, thyroid nodularity; thrombocytopenia;
Patient 9	28	55	64	3	G1P0	Antepartum	Lymphadenopathy and splenomegaly NDY, chronic headache, borderline high blood pressure, overweight, Herpes Zoster (early pregnancy), endometriosis
Patient 10	28	160.2	108	1	G2P2	Postpartum	Pulmonary embolism, postpartum anemia secondary to postpartum hemorrhage; irritable bowel syndrome, PTSD;
Patient 11	22	56	64	1	G1P1	Postpartum	Episodic hypotension; arrhythmia
Patient 12	41	30.6	44	2	G2T0A1L0	Antepartum	Pre-existing/chronic hypertension; iron deficiency anemia;
Patient 13	37	19.2	30	1	G2T1P1A0L1	Postpartum	Allergic rhinitis, asthma, acid reflux
Patient 14	40	6.4	16	4	G4P2A2	Postpartum	Type 1 diabetes; postpartum hypertension
Patient 15	36	168	130	1	G4T1A2L1	Postpartum	Pulmonary embolism

**Fig. 1:**
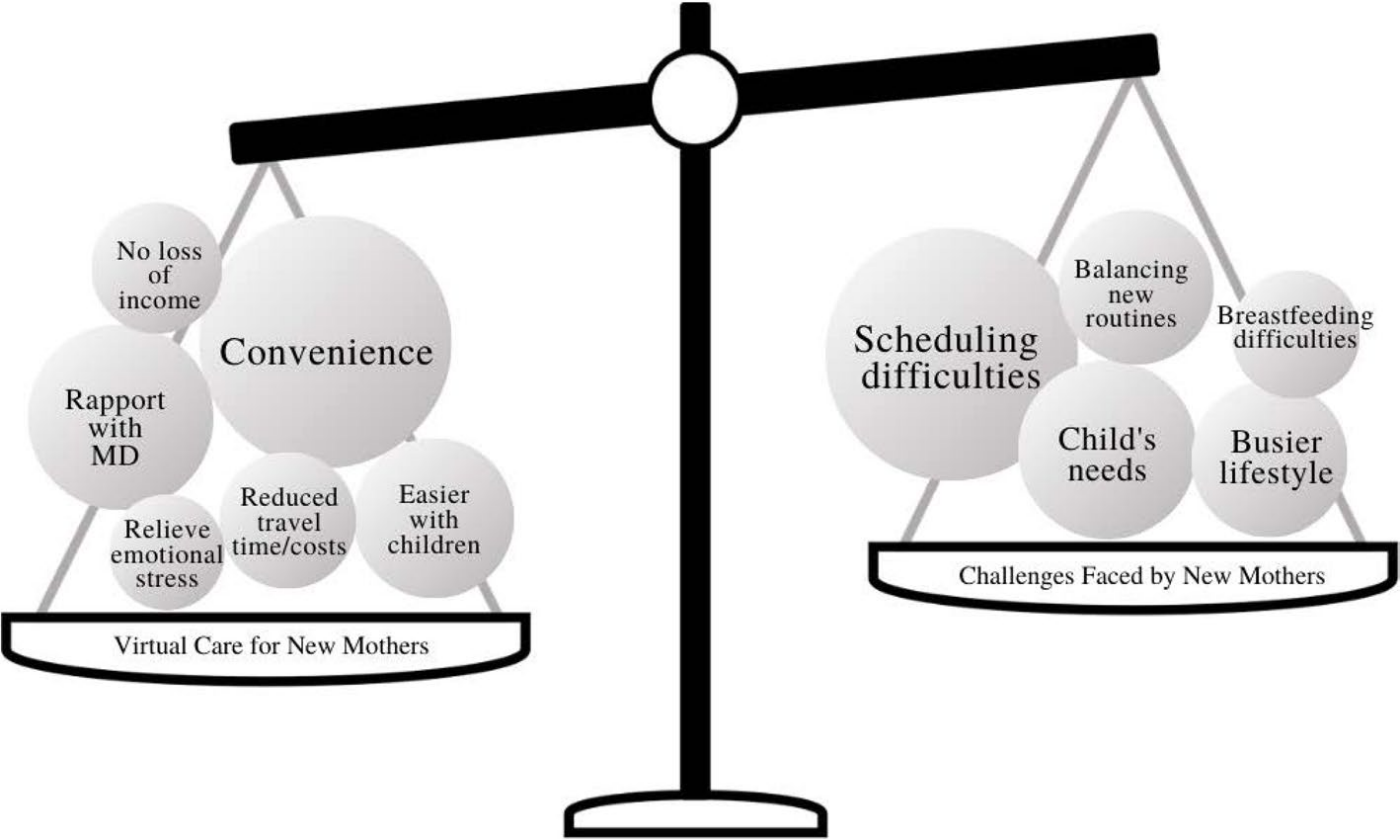
Two broad umbrella themes that emerged through second stage coding

**Fig. 2:**
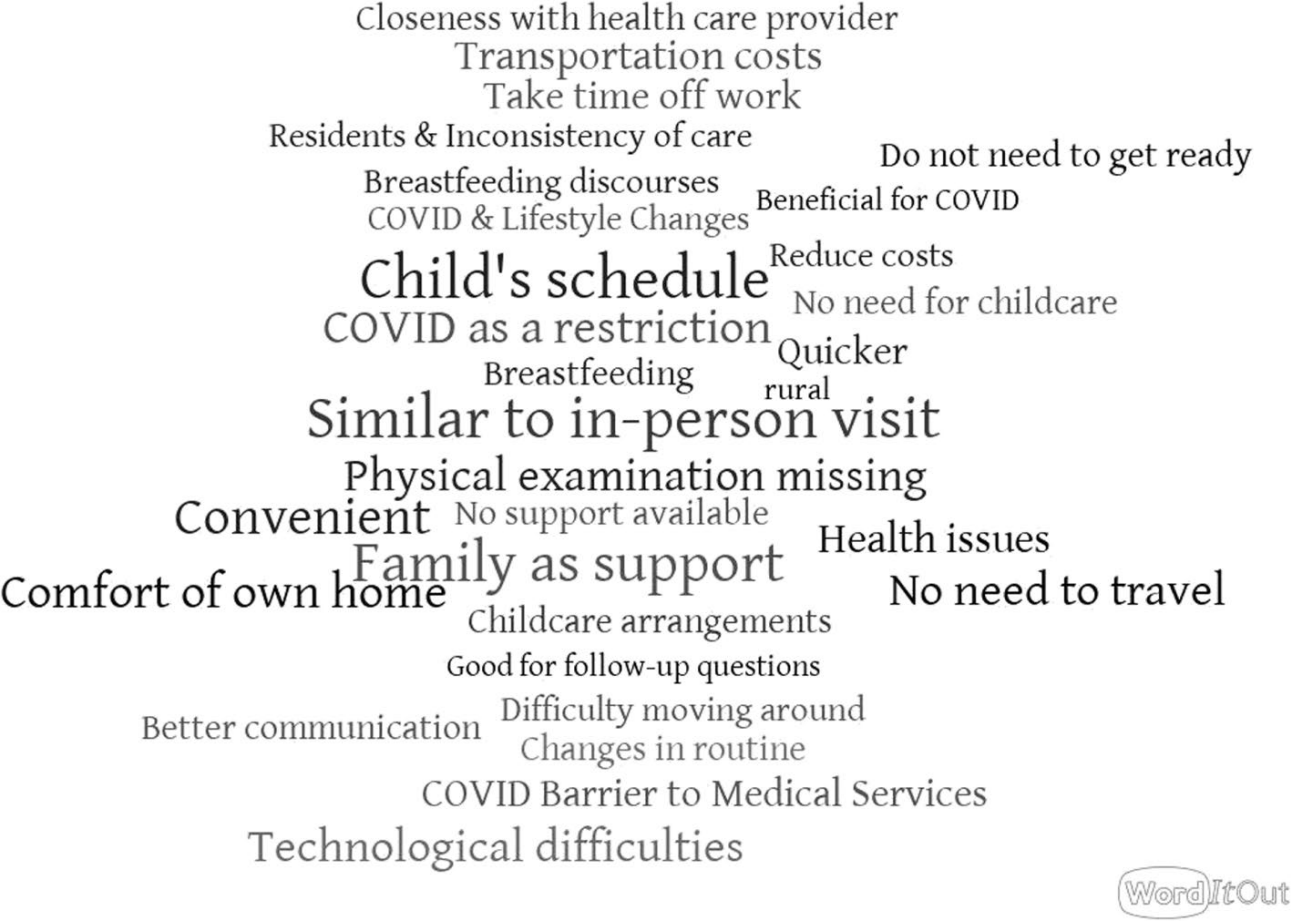
Word map of the 30 most frequently occurring themes in the study. The presented themes in this word map were referenced at least 2 times

As per the objective of the study to understand the perceptions of new mothers using virtual care, we identified two major themes: benefits and challenges of using virtual care (Fig. 1). We found three subthemes under the theme of “benefits”: increased comfort and convenience; relieving emotional and financial stress; and facilitating emotional support and rapport with the physician (Fig. 2). Participants perceived that lack of familiarity with technology use was a major barrier to virtual care use.

### Benefits of Using Virtual Care

#### Increased comfort and convenience

Across the interviews, participants spoke broadly about their mothering experiences. Many participants spoke specifically to the challenges of being a new mother and changes they have adopted to their daily lives since having a child. Some of the changes included having to prioritize their child’s needs, adopting new routines, and balancing multiple conflicting priorities. Challenges associated with these changes included scheduling difficulties for healthcare appointments or other daily activities, increased demands for planning and arranging the day, difficulty breastfeeding, time constraints associated with a busier lifestyle, and the overall burden of care on new mothers. Table 2 documents the travel time and distance that our participants had to take to participate in the in-person clinic. The mean travel time for all participants to drive from their home to the hospital and back to their home was 78.3 min. Participants would spend much more time travelling if they had multiple visits.

Participants perceived that virtual care facilitated comfort and convenience which included the ability to stay at home to conduct the call and to work around the schedule of the participants. Many participants perceived that virtual care could be a useful modality as it provided patients an opportunity to receive care while balancing the domestic tasks of new mothers. Some of these tasks included working around their child’s eating and sleeping schedule, accommodating for the schedules of multiple children in the household, or working around the schedules of their caregivers or family members. Further, participants with co-morbidities such as chronic migraines and irritable bowel disorder could make it difficult to travel long distances. All participants reflected positively on their experiences with virtual care:

*It’s great because I don’t have to worry about getting ready and making child care arrangements. I can just put my son in the chair next to me and it makes it far more accessible. Neither of us has to take time off work really (P3, aged 34).*

*It made it a lot easier ‘cause it was a lot less time consuming, not having to pack up and go out. I can even do it while the baby was napping and not have to mess up their schedule, like, kids’ schedules. It was a lot easier (P4, aged 34).*

*I found it much easier to just be able to be at home, not have to worry about getting the kids ready and long care rides or have to worry about findings someone to watch them. They were very good if I needed to take care of the baby for a second or breastfeed. There’s a lot you do (P9, aged 28)*.

#### Relieving stress associated with in-person care

Participants identified that travel time, distance, finding parking, and cost were barriers to attending in-person obstetric care, leading to undue stress. Participants perceived that virtual care could be a useful modality to relieve financial and emotional stress related to attending the hospital.

*You didn’t feel a disconnect. I was able to speak freely with [obstetrics physician] and in the comfort of my own office. I have a door, I was able to shut the door and just have the meeting during my lunch hour so it was perfect (P12, aged 41)*.

*Saving gas money, parking money, and I hate driving so it was a huge emotional stress driving to Kingston every single time. My mental health was much better for not having to go at that time (P8, aged 31).*

*I didn’t have to worry about either being off work or being on lunch or making sure I could leave work or whatever (P2, aged 35).*

#### Facilitating emotional support and building rapport with the physician

Lastly, participants perceived that virtual care gave them easier access to emotional support, helping them to build rapport with their obstetric medicine physician.

*Kingston’s a far drive for me so when I was near the end, especially when you’re heavily pregnant, I already had that emotional support and I could trust my doctor, so just being able to do a virtual visit was huge for me. It helped my husband not have to arrange his schedule for work and it helped me still stay reassured that everything was going to be fine (P8, aged 31).*

*As a mom, you have so many questions about is this normal, is this working right and you don’t really know who to turn to with those questions. I’m quite a worrier so I worry about a lot of things. It’s just nice to have that. It’s a little less formal. You don’t feel like you’re taking up a lot of time. You don’t have to book an appointment just to get one quick question answered (P1,* aged *36).*

### Challenging Aspects of Using Virtual Care

Few participants spoke to the negative aspects of using virtual care. While many of the participants in this study did not experience technological difficulties, some perceived that poor internet connectivity, audio issues, and technology could serve as barrier for some patients, especially those in rural communities or those who do not have access to high-speed internet:

*For me it’s easy to navigate. If it was my parents it would be a nightmare. They’re…needs to be seen in person. It’s just not gonna happen. Accessibility, we’re in a rural area and we don’t really even have high speed internet. We’re lucky we’re still not dial-up…My parents live in a place where it’s hard to even get any internet. They live five minutes down the road and you have to pay for one of those special satellite things to come or get the tower in order to even get internet (P8, aged 31).*

*I come from an area, if you drive five minutes down the road, you can’t even really get access [inaudible]. It’s terrible. (P12, aged 41)*

## Discussion

The purpose of this study was to understand the perceptions of new mothers (obstetric patients) using virtual care for postnatal follow-up care. The data suggests that virtual visits were considered a convenient, low-barrier, and accessible modality for healthcare delivery for postnatal follow-up care. In the context of being a new mother, participants felt that virtual visits were able to address many challenges unique to this population, such as scheduling difficulties, needing to arrange for childcare, and time constraints due to busier routines. These findings are consistent with previous literature, where patients found virtual care to be convenient, time-saving, and accessible for patients with unique physical impairments [15, 26].

### Benefits of virtual care as a means towards patient compliance to follow-up care

#### Sub-Theme 1: Increased comfort and convenience

While there have been relatively limited studies examining the use of virtual care in the form of video conferencing for maternal healthcare in the postpartum period, our results are consistent with previous literature examining patient satisfaction with virtual obstetric care in the prenatal period [27]. Virtual visits have been found not only be convenient, but to uphold the same level of rapport in the patient-provider encounter. In a U.S.-based cross-sectional survey examining patient’s preferences for telehealth visits, almost 1/3^rd^ of participants expressed a preference for telehealth, and over ½ of participants rated telehealth to be just as good as traditional in person care [28]. Likewise, our participants expressed that virtual visits were comparable or even better than in-person visits where follow-up do not necessitate being seen for a physical examination.

The participants in this study also expressed enthusiasm for the use of virtual visits in the follow-up care context, where the convenience of being able to see a provider at home for a simple follow-up appointment was significant in terms of comfort, convenience, and costs. In our study, several participants recalled that they had forgotten to attend their appointments. However, all felt inclined to use virtual visits once they were reminded to do so through phone call by the clinic secretary due to its perceived ease of use. This is consistent with literature examining patient experiences with telehealth for follow-up care specifically, and further supports the utility of virtual care for increasing compliance to postnatal follow-up healthcare appointments [29].

In other studies of the use of telemedicine in early discharge from childbirth, patients also reported feeling confident with the ease of the technology and did not perceive a threat to their privacy or personal information. In a pilot project based in Sweden, patients reported feeling confident that their concerns and questions were addressed to the same degree through video conferencing and that communication with their provider was not compromised [30]. This may be attributed to the fact that younger mothers may already incorporate technology in their daily lives [31]. Resultantly, young mothers may more positively take up virtual care in comparison to other patient populations or age groups.

#### Subtheme 2: Relieving stress associated with in-person care

Our participants also expressed how virtual visit was able to address barriers related to financial constraints, distance, travel time, and arranging time off work, which is consistent with previous literature examining the benefits of virtual care visits in primary care settings. In our study, virtual care was found to be highly favored amongst participants living out of the city and/or in rural towns, as they did not have to spend time, costs, and energy to attend to the hospital. Virtual care was also found to be useful for women with multiple children in our study, as they were not required to make extra childcare arrangements, find caretakers, and spend associated costs. Other participants perceived that virtual care would not interrupt their source of income as they were able to conduct the virtual session otherwise. This was particularly relevant throughout the ongoing COVID-19 pandemic, as patients were not permitted to bring other children to in-person visits in order to prevent unwanted exposure. These findings are also consistent with other studies exploring the use of virtual care in other patient populations, which support its use in alleviating the aforementioned barriers related to accessing in-person healthcare services [13, 15, 26, 32].

Overall, the perceived benefits of virtual care could provide insight into ways to increase patient compliance to post-natal follow-up care. In another study conducted by researchers at the Kingston Health Sciences Centre, it was found that increasing distance from the hospital was significantly associated with poorer attendance at follow-up visits. To a less significant degree, it was also found that poorer follow up was associated with patients with a greater number of living children and women with no educational certificate, diploma, or degree [10].

#### Subtheme 3: Facilitating emotional support and rapport with the physician

Rapport and trust is important to building strong relationships between physicians and patients. Rapport is also an important part of providing compassionate care and emotional support, particularly as new mothers experience physiological, emotional, and domestic changes related to childbirth [33]. However, it may be more difficult to establish rapport and provide support without in-person interactions. Most of the available literature on virtual care has focused on patient experiences more generally, and less on examining patient-physician rapport in virtual care.

Our findings illustrate the significance of building strong rapport between patients and their obstetric physician. Participants felt they were able to feel “calm” and supported by their obstetric physicians because they were able to contact their physician at their convenience when they had a question. Thus, participants perceived that virtual care by means of video conferencing was particularly useful as they were able to receive affirmation by seeing the gestures and demeanor of their physician. In this way, participants were able to build trust and rapport with their physician despite receiving care remotely. Given the significance of building rapport and trust for obstetric patients, physicians and patients would benefit from more research on ways to build rapport through video-based virtual care platforms to improve long-term compliance and patient-centered care.

### Barriers of virtual care and the need to consider social factors and determinants of health

Virtual care appears to be an important modality for increasing compliance to post-natal follow-up care. This is likely due to the fact that virtual care addresses the various social and environmental factors that intersect to impact health care access. Income, geography, and educational background are commonly understood as some of the most significant social determinants of health [18, 34, 35]. Compounded by domestic tasks and expectations, women may feel overwhelmed, overburdened, or disinclined to attend postnatal follow-up clinics [36]. Since virtual care can be conducted at a place comfortable for the patient and at their own schedule, it may become easier for women to attend to attend regular follow-up visits for maternal health monitoring. As a result, virtual care should be positioned not only as a tool to facilitate compliance in follow-up, but as a tool to evaluate the various social and environmental determinants that may encourage or deter patients from attending follow-up clinics.

Our study did allude to one major limitation of virtual visits, in that the internet connection can vary considerably between patients and pose a potential barrier for conducting virtual care appointments [37]. This limitation can be exacerbated by various socioeconomic factors such as rurality or financial constraints. Patients who are unable to afford a high-speed internet connection or those who live in rural areas with poor connectivity may find themselves limited in their ability to access virtual care appointments [26]. This finding is consistent with previous literature indicating that virtual modalities for healthcare delivery in primary care can be limited when it comes to connection issues [38–42].

### Limitations of the study

This study was limited by the relative homogeneity of the sample. As many of the participants were white/Caucasian, married, Canadian-born, middle-class, and well educated women, the perceived positive and negative aspects of virtual visits could be different if a more heterogenous sample was interviewed. For example, as Canada is an immigrant-dense country, language barriers, especially if virtual visits are not offered in multiple languages, could potentially be a limiting factor. However, participants were highly satisfied with the virtual visit platform for postnatal follow-up appointments, and the comfort and convenience it provides to participants are relevant across patient demographics. The research team also acknowledges that the sample size for this study is relatively small. This could be attributed to a large number of patients who had declined to participate as well as the research team’s decision to take an ethnographic approach to the data in order to draw out rich details from the participants. The research team also acknowledges that a potential source of selection bias is that the obstetric medicine physicians on the team conducting the virtual visits asked patients about their interest in study participation. Those study team members invited interested patients to participate in the study. There is also potential selection bias as only some women from the clinic participated in the virtual visits. However, this form of purposive sampling was necessary because 1) there was only a small sample available, and 2) because virtual care was relatively new at KHSC at the time of the study, not all patients received it during the study period.

## Future directions and conclusion

While this study showed that new mothers found virtual visits to be a suitable modality of healthcare delivery for postnatal follow-up, our interviews alluded to a variety of challenges that new mothers experience that may differentially impact their ability to seek care. Therefore, future studies should investigate barriers to accessing in-person healthcare services for new mothers in order to better understand the unique determinants and barriers of this patient population. Intersectionality theory would serve well to illuminate the connections between the social determinants of health and barriers to virtual care. Furthermore, virtual care has the potential to significantly shift the landscape of healthcare delivery in the COVID era, and further research should specifically examine patient experiences of virtual visit in relation to the ongoing pandemic.

In conclusion, qualitative interviews with new mothers who have used virtual care in the form of virtual visit for postnatal follow-up appointment visits revealed high satisfaction with virtual care, emphasizing benefits in terms of comfort, convenience, communication, socioeconomic factors, and the ease of technology use. In the follow-up care context, our findings suggest that virtual care increases accessibility and ease of follow-up care for patients with unique barriers to accessing healthcare services. Such a lens not only addresses the struggle of long-term patient compliance to maternal health care, but will also shed light on how to make obstetric care more equitable. Future directions should seek to examine the barriers to accessing in-person healthcare services unique to new mothers and examining the applicability of virtual visits for this patient population in the context of the ongoing COVID-19 pandemic.

## Supplementary Information

**Additional file 1.**

## Data Availability

As per the conditions with the Queen's University's Health Sciences Research Ethics Board, the datasets generated during and/or analysed during the current study are not publicly available because of the chance of identification due to a small sample size. However, the data can be made available from the corresponding author on reasonable request.
