# Supplementary material for: Patient perceptions of the benefits and barriers of virtual postnatal care: a qualitative study
Source: BMC Pregnancy Childbirth. 2021 Aug 7;21:543. doi: 10.1186/s12884-021-03999-9 (PMC8346781; doi:10.1186/s12884-021-03999-9)
Supplement: Supplementary file 1 — Additional file 1. [file 12884_2021_3999_MOESM1_ESM.docx]

**Title:** Patient perceptions of the benefits and barriers of virtual postnatal care: A qualitative study

**Authors:** Megan Saad^1^, Sophy Chan^,2^, Lisa Nguyen^2^, Siddhartha Srivastava^2^, Ramana Appireddy^2^

**Affiliations:** ^1^School of Medicine, Queen’s University, Kingston, Ontario, Canada; ^2^Department of Medicine, Queen’s University, Kingston, Ontario, Canada,

**Corresponding Author:** Dr. Ramana Appireddy

Department of Medicine, Queen’s University

94 Stuart Street

Kingston, ON, Canada, K7L 3N6

Email: [mrra@queensu.ca](mailto:mrra@queensu.ca)

Tel: 613 549 6666 ext 6119

**Keywords:** Telemedicine, Postpartum Period, Postnatal Care, Qualitative Research, Social Determinants of Health

**Obstetrics E-Visit Semi-Structured Interview Guide**

**PERSONAL BACKGROUND**

To start, please tell me a little bit about yourself.

- SPECIFIC QUESTIONS: What is your profession? What work do you do? Do you live nearby and did you grow up here?

(If not addressed above) I am now going to ask you a few more follow-up questions to get to know more about you. Once again, I’d like reiterate you do not have to answer any questions you do not feel comfortable.

- What ethnicity(ies) do you most identify with?
- Were you born in Canada? If not, when did you move here and from what country?
- What is the highest educational degree you have earned?
- What is your current marital status?
- What gender do you most identify with? For example, woman, man, non-binary, transgender, etc.
- Can you tell me under which letter your household income falls under?:
  1. $0 to $30,000
  2. $30,000 to $59,999
  3. $60,000 to $89,999
  4. $90,000 to $119,999
  5. $120,000 to $149,999
  6. $150,000 or more
  7. Prefer not to answer
  8. Do not know

Since having your child, how has your life or day-to-day routine changed?

- FOLLOW UP PROMPT
  - What are some of the provisions or changes you have made to your life since having your child?

**1) IN-PERSON HEALTHCARE**

In this section, I will ask you questions about your experiences accessing healthcare in person, for example at the hospital or at your family doctor’s office.

**BARRIERS AND FACILITATORS TO IN-PERSON CARE**

What are some barriers you have experienced that have limited your ability to access healthcare in person?

- FOLLOW UP PROMPTS
  - How much does it cost you to come for an in-person visit including the loss of pay, travel, parking, childcare, or for a caregiver or family member to accompany you, etc?
  - Do you provide care to anyone else in your household?
  - Are there any other factors that have limited or stopped your ability to access healthcare in person?

What sort of arrangements do you have to make at home/work to see a doctor for follow-up visits?

- FOLLOW UP PROMPTS
  - How much time do you spend during an in-person visit including the time needed to prepare to leave home/work, travel time, parking arrangements, registration, waiting, actual time with the physician and nurses, residents and medical students?

What are some factors that you have experienced that have made it easier for you to access healthcare in person?

- FOLLOW UP PROMPTS (For example, support from your partner, maternity leave, etc.)
  - How easy is it for you to schedule your appointments?
  - How has your partner or other supports made it easier for you to access care in person?

**RELATIONSHIP WITH PROVIDER**

How has your relationship with your healthcare provider affected your experience accessing in person care?

- PROMPT: For example, friendliness/courtesy provider, concern for your questions, sensitive to your needs etc.

**FAMILY/CAREGIVER INVOLVEMENT**

What do you think is the impact of family on someone’s ability to access healthcare in person?

What do you think is the impact of friends on someone’s ability to access healthcare in person?

What do you think is the impact of social support networks on someone’s ability to access healthcare in person?

**ACCESSING SERVICES**

From what you know, can you describe what medical and social services are available to you as a new mother?

What services do you access and why?

**2) VIRTUAL CARE**

In this section, I will ask you questions about your experiences accessing virtual healthcare through eVisits, and how that compares to your experience accessing care in person.

**BARRIERS AND FACILITATORS TO VIRTUAL CARE**

Has eVisit addressed any of the barriers that you experience in accessing in-person healthcare? If so, how?

Has eVisit made it easier for you to access healthcare? If so, how?

**RELATIONSHIP WITH PROVIDER**

How does your relationship with your healthcare provider and/or staff compare from in person care to in person care?

FOLLOW UP PROMPTS

- Have you felt that your concerns have been heard? Have you felt that your health care provider has been able to assess you to your satisfaction?
- Were you able to build rapport and communicate effectively with your physician?
- Did the degree to which the provider took time to listen to you change? Did they show the same concern for your questions? Were they sensitive to your needs?

**FEASIBILITY AND EASE OF TECHNOLOGY**

Where do you usually conduct your eVisit appointment?

What technology do you use for your eVisit? (ie. Smart phone, iPad, etc.)

- FOLLOW-UP PROMPTS
  - Do you own this equipment or is it borrowed?
  - Are you able to use this technology without help?
  - Is the technology easy for you to use?
  - Do you have someone who can help you with the technology?

Following up on this, are there any aspects of e-Visit that concern you? (For example, issues of security, not receiving proper care, the technological aspects are difficult to navigate)

**OVERALL EXPERIENCE**

Overall, how was your experience using eVisit instead of a face-to-face follow-up visit?

- FOLLOW-UP PROMPTS
  - Were there any other differences between your in-person visit and eVisit? If yes, did these differences affect the quality and/or effectiveness of your eVisit?
